# Development and Comparison of Complementary Methods to Study Potential Skin and Inhalational Exposure to Pathogens During Personal Protective Equipment Doffing

**DOI:** 10.1093/cid/ciz616

**Published:** 2019-09-13

**Authors:** Jennifer Therkorn, David Drewry, Jennifer Andonian, Lauren Benishek, Carrie Billman, Ellen R Forsyth, Brian T Garibaldi, Elaine Nowakowski, Kaitlin Rainwater-Lovett, Lauren Sauer, Maggie Schiffhauer, Lisa L Maragakis

**Affiliations:** 1 Applied Biological Sciences, Johns Hopkins Applied Physics Laboratory, Laurel; 2 Department of Hospital Epidemiology and Infection Control, Johns Hopkins Health System, Baltimore, Maryland; 3 Johns Hopkins University School of Medicine, Baltimore, Maryland; 4 Johns Hopkins Bloomberg School of Public Health, Baltimore, Maryland

**Keywords:** personal protective equipment doffing, exposure assessment, doffing self-contamination, inhalational exposure, methods development

## Abstract

**Background:**

Fluorescent tracers are often used with ultraviolet lights to visibly identify healthcare worker self-contamination after doffing of personal protective equipment (PPE). This method has drawbacks, as it cannot detect pathogen-sized contaminants nor airborne contamination in subjects’ breathing zones.

**Methods:**

A contamination detection/quantification method was developed using 2-µm polystyrene latex spheres (PSLs) to investigate skin contamination (via swabbing) and potential inhalational exposure (via breathing zone air sampler). Porcine skin coupons were used to estimate the PSL swabbing recovery efficiency and limit of detection (LOD). A pilot study with 5 participants compared skin contamination levels detected via the PSL vs fluorescent tracer methods, while the air sampler quantified potential inhalational exposure to PSLs during doffing.

**Results:**

Average PSL skin swab recovery efficiency was 40% ± 29% (LOD = 1 PSL/4 cm^2^ of skin). In the pilot study, all subjects had PSL and fluorescent tracer skin contamination. Two subjects had simultaneously located contamination of both types on a wrist and hand. However, for all other subjects, the PSL method enabled detection of skin contamination that was not detectable by the fluorescent tracer method. Hands/wrists were more commonly contaminated than areas of the head/face (57% vs 23% of swabs with PSL detection, respectively). One subject had PSLs detected by the breathing zone air sampler.

**Conclusions:**

This study provides a well-characterized method that can be used to quantitate levels of skin and inhalational contact with simulant pathogen particles. The PSL method serves as a complement to the fluorescent tracer method to study PPE doffing self-contamination.

## Teaser

A new method is pilot tested against the fluorescent liquid tracer method for quantifying skin and inhalational exposure to pathogens during personal protective equipment doffing. The new method uses fluorescent polystyrene latex spheres to simulate pathogen particles.

In September 2015, 2 healthcare workers at Texas Health Presbyterian Hospital became infected with Ebola virus disease (EVD) after providing care for a patient infected with Ebola virus [1, 2]. This case raised issues regarding whether the appropriate protections were provided to staff members, including training for safe and effective use of personal protective equipment (PPE), particularly the PPE doffing process. Proper PPE doffing is vital to reduce the risk for self-contamination and is an area of focus to ensure healthcare worker safety [3–7].

Given the risk of self-contamination during PPE doffing procedures, it is important to assess the likelihood of contamination at each point of the process. Furthermore, enhanced PPE ensembles for care of patients with high-consequence pathogens have multiple layers, adding to the complexity of doffing practices and increasing the risk of self-contamination [2, 6, 8–10]. Commercially available fluorescent products (liquid, lotion, or powder) used as tracers in combination with ultraviolet (UV) lights are frequently used as a study method to detect PPE doffing self-contamination [3, 6, 11, 12]. This approach provides feedback to healthcare workers as they can visualize where they have contaminated themselves [3]. However, this detection method has limitations. It has a relatively low sensitivity of detection, as eyesight alone cannot identify pathogen-sized fluorescent particles that may constitute contamination. Second, the commonly used fluorescent powders that dissolve into a liquid suspension may not fully or accurately represent the full spectrum of exposure risks. Specifically, recent research has shown that there is the potential for reaerosolization of infectious particles from PPE during doffing [13], and this type of potential inhalational exposure has not been addressed by the fluorescent tracer method.

This study presents the development of a more sensitive method to investigate self-contamination using fluorescent, bacteria-sized (2 µm) polystyrene latex spheres (PSLs); additionally, for the first time, potential inhalational exposure for healthcare personnel during PPE doffing is investigated with the use of an air sampler to quantify PSLs in the doffer’s breathing zone. This approach is then directly compared to the traditional fluorescent tracer method in a pilot study to investigate self-contamination levels using the Centers for Disease Control and Prevention (CDC) PPE doffing guidelines for providers caring for patients with EVD or other high-consequence pathogens [14].

## METHODS

This study encompasses 2 main components: (1) PSL contamination and detection methods development; and (2) a PPE doffing pilot study to evaluate the PSL method vs the fluorescent tracer method (Table 1). PSLs are a nonhazardous, inert substance. More background information on the approach and rationale for selection of the PSLs can be found in Drewry et al [13].

**Table 1.** Comparison of Polystyrene Latex Spheres and Fluorescent Tracer Methods for Studying Self-contamination During Personal Protective Equipment Doffing

Method	PSL Method	Fluorescent Tracer Method
Method description	2-µm PSLs (G0200, Thermo Fisher Scientific, Waltham, Massachusetts) diluted in water	Fluorescent powder slurry (Glitter Bug, Brevis Corporation, Salt Lake City, Utah) mixed in a viscous suspension of water and oil
Suspension composition	PSLs diluted 1:10 in filtered, deionized water (10^9^ PSL/mL)	Glitter Bug powder (75 mg/mL) mixed in grapeseed oil and water (1:6 ratio oil to water)
Application of contaminant to healthcare worker study subjects wearing PPE	25 mL of PSL suspension in 3-jet Collison nebulizer (Mesa Laboratories, Butler, New Jersey); 4 min of continuous aerosol generation while healthcare worker turned 90º every 60 sec	1000 mL of mixture in a pesticide hand sprayer (RL Flo-Master 2000 mL capacity, Lowell, Michigan); 5 sweeping passes of sprayer from head to feet on front and back of healthcare worker
Simulated contamination type	Representative of pathogen dispersal via respiratory secretions, such as coughing and sneezing [13, 15]	Representative of a wet patient and gross liquid contamination [6]; the grapeseed oil was included to represent a viscous bodily fluid
Sampling method	From skin: sterile foam-tipped swabs (13-cm handle, scored with thumb stop, Puritan Medical Products, Guilford, Maine) premoistened in filtered, deionized water with 0.05% Triton-X 100	Not applicable
	From air in breathing zone: Button Sampler (SKC, Eighty Four, Pennsylvania), operated at 4 L/min with 25 mm PTFE (polytetrafluoroethylene) filters of 3-µm pore size (catalog number 225–1711, SKC) [16]	
Detection method	PSL counting via epifluorescent microscopy	Visual inspection with an ultraviolet lamp
Quantification method	Skin: number of PSLs per cm^2^ of skin on each swabbed body part	Number and relative size of contamination spots detected on skin and scrubs
	Button Sampler: number of PSLs per m^3^ of sampled air	

Abbreviations: PPE, personal protective equipment; PSL, polystyrene latex sphere.

### PSL Protocols: Skin Swabbing and PSL Quantification by Microscopy

To establish the limit of detection for the PSL method, known quantities of PSLs were pipetted onto and then sampled from porcine skin coupons using swabs, and the full procedural recovery efficiency was estimated (ie, from skin swabbing to elution of PSLs from the swab tip). Given the potential for procedural loss of PSLs from skin swabbing, this experiment with skin coupons allowed for determination of the correction factor to use in the pilot study doffing experiments to scale up the observed numbers of PSLs recovered from skin swabs to estimate the actual total number of PSLs present on skin.

The process used 4500 g of porcine skin, a human skin substitute [17–19], with a skin thickness of about 1.5 cm. The skin was cut into rectangular-shaped coupons (50 cm^2^) using a scalpel and cleaned with 70% ethanol followed by a disinfecting wipe (Oxivir wipes, Diversey, Charlotte, North Carolina). The disinfectants were allowed to dry on the skin coupons, and then they were put in plastic zip bags and frozen (−20°C) until use. The coupons were removed from the freezer and thawed at 4°C for 24 hours prior to use.

The PSL solution was serially diluted to achieve concentrations of 10^2^–10^6^ PSL/mL. Using the methods of Therkorn et al [20], the 5 PSL concentrations were spiked (100 µL) onto the skin coupons, with each dilution spiked on 5 different skin samples (25 total coupon samples). For each of the spiked PSL concentrations, there were 5 control standards prepared where 100 µL was pipetted directly into 1 mL of filtered, deionized (DI) water.

After allowing the spiked droplets to dry for 5 hours in a biosafety cabinet, each skin coupon was swabbed to collect PSLs. Each coupon was swabbed with 1 sterile foam tipped swab premoistened in an elution suspension of filtered deionized water with 0.05% Triton-X 100 as previously recommended in similar research [21–23]. Each swab was assigned a 15-mL conical tube containing 5 mL of the elution suspension. Swabs were dipped into the elution suspension, and excess fluid was expressed from the swab by pressing against the inside of the tube. The moistened swab was then swept across the entire surface of the skin coupon 4 times in the following directions: horizontal, vertical, diagonal from top right to bottom left, and opposite diagonal direction [24]. The swab was turned each time the sweeping direction changed to expose all surfaces of the swab. Then, the swabs were placed back into the elution fluid in the conical tube and stored at 4°C until processing. As recommended, only 1 person conducted all of the swabbing to minimize sampling variability [25–27]. To ensure there was no contamination introduced onto the coupons in any of the procedures, 3 clean skin coupons were swabbed to serve as blanks.

The PSLs were eluted from the swabs using a 2-minute vortex followed by a 10-minute ultrasonication (M1800, Branson Ultrasonics, Danbury, Connecticut) [20, 28]. PSL detection and counting were conducted using a modified version of the Collection of Airborne Microorganisms on Nucleopore filters, Estimation and Analysis method [20, 29] via epifluorescence microscopy (Nikon Eclipse Ni-E, Nikon Instruments, Melville, New York). A 1-mL volume of elution suspension containing PSLs was filtered through a black polycarbonate filter (GTMP, 0.2 µm, 25 mm, Merck Millipore, Cork, Ireland). The filter was allowed to dry and then fixed onto a microscope slide. At least 40 random field views were counted and averaged, and this averaged number was multiplied by the total number of field views to estimate the number of PSLs across the entire filter [29]. To determine the PSL recovery efficiency and limit of detection, the results were then calculated as the recovered number of PSLs per swab relative to the actual spiked number of PSLs as determined from the prepared standards.

### Air Sampling for PSLs in the Subjects’ Breathing Zone

An additional sampling method was employed to assess the possibility of exposure to aerosolized PSLs during the doffing process. The Button Sampler, a commonly used personal breathing zone sampler in occupational and environmental health studies [30–33], was used to measure the concentration of airborne PSLs in the subjects’ breathing zone during PPE doffing. This sampler closely follows the convention for sampling inhalable airborne particles [34]. The Button Sampler was clipped onto the shirt collar of the scrubs underneath the PPE, and the air pump of the sampler was attached to the powered air purifying respirator (PAPR) belt. Care was taken to ensure that the tubing from the Button Sampler to its pump did not interfere with the PPE placement and did not change the wearer experience. The sampler pump was turned on for collection prior to the subject entering the room for contamination. The sampler on/off button was easy to push through the PPE so as not to disturb the ensemble. The pump ran continuously throughout contamination and doffing to measure potential inhalational exposures during the process. It was turned off by the trained observer after the final step in the doffing process. The filter inside the Button Sampler where the PSLs were captured was recovered with clean forceps and stored in a 50-mL conical tube for each subject. The PSLs were eluted from the Button Sampler filters and enumerated using the same methods as described above for the swabs.

### Pilot Test

A pilot test was conducted to compare the results of the PSL method to the fluorescent tracer method. The CDC’s PPE donning and doffing guidelines for care of patients with EVD were used for this pilot test [14]. Table 2 describes the elements of the PPE ensemble. Five subjects were recruited to participate who had no prior experience with the PPE and doffing guidelines. The study protocols were approved by the Johns Hopkins School of Medicine Institutional Review Board (approved 26 January 2018, IRB00084233). All participants provided written informed consent as required by the IRB protocol.

**Table 2.** Elements of the Personal Protective Equipment Ensemble Tested in the Pilot Study

PPE Item	Product Name	Manufacturer
Gown	SmartGown impervious surgical gown	Cardinal Health (Dublin, Ohio)
Gloves (outer)	Biogel Skinsense (synthetic [nonlatex] polychloroprene surgical glove)	Mölnlycke (Gothenburg, Sweden)
Gloves (inner)	Synthetic (nonlatex) polychloroprene surgical glove (synthetic [nonlatex] polychloroprene surgical underglove)	Mölnlycke
Isolation gown	MediChoice overhead poly-coated gown	Owens and Minor (Richmond, Virginia)
Boot covers	Hi Guard regular full coverage boot, universal size	Kimberly Clark (Irving, Texas)
Belt-mounted high efficiency PAPR	Air-Mate Assembly 231-01-30	3M (Maplewood, Minnesota)
Tape	Duct tape	3M
PAPR hood	White Respirator Hood BE-10–3 (regular, Tychem double shroud)	3M

Abbreviation: PAPR, powered air purifying respirator; PPE, personal protective equipment.

**Figure 1.**
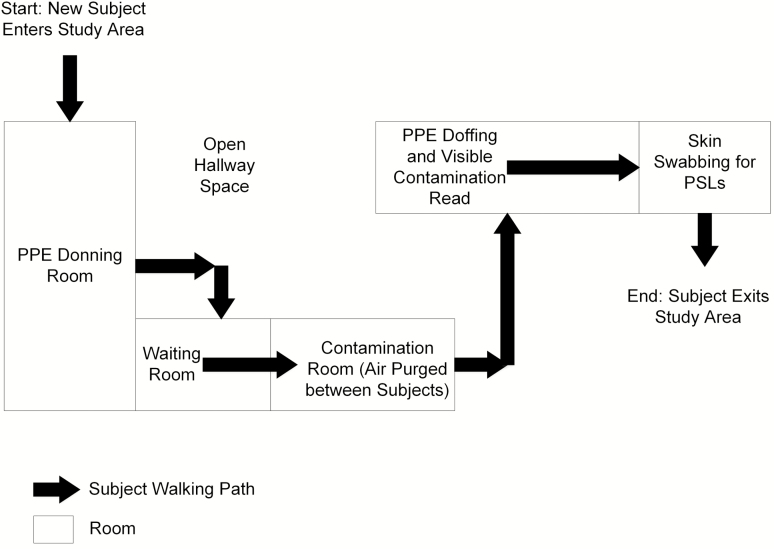
Map of the study area indicating subject walking path and separate rooms for each activity: (1) personal protective equipment (PPE) donning; (2) contamination; (3) PPE doffing and visible (Glitter Bug) contamination check; and (4) skin swabbing for polystyrene latex spheres. Abbreviations: PPE, personal protective equipment; PSLs, polystyrene latex spheres.

All subjects were provided paper scrubs and hospital-approved footwear and asked to remove any accessories. Each subject wiped his or her face with a skin cleansing wipe to remove makeup and excess skin oil (Gentle Skin Cleansing Cloths, Cetaphil, Galderma, Fort Worth, Texas). The study then proceeded as follows (detailed description below): (1) study subjects were checked by investigators for background fluorescent contamination on skin and scrubs in a dark room under UV light; (2) the Button Sampler was attached to the shirt collar of scrubs; (3) PPE was donned by subjects; (4) subjects were contaminated with both the fluorescent tracer and the PSLs; (5) PPE was doffed by subjects; (6) subjects were checked for fluorescent tracer contamination by UV light; and (7) subjects were swabbed for PSLs and Button Sampler filters were retrieved. To minimize any potential for unintended cross-contamination of subjects during the experiment, separate rooms were used for PPE donning, subject contamination, PPE doffing, and skin swabbing, with a designated walking path between these rooms (Figure 1).

After subjects donned their PPE according to the CDC guidelines for the care of patients with EVD, they were then contaminated with the 2 different materials: fluorescent tracer slurry followed by nebulized PSLs. Table 1 provides details on these 2 methods, including suspension composition and equipment used for spraying the contamination. Participants were instructed to stand with palms facing forward on a marked spot on the floor in the contamination room 0.5 m away from where the fluorescent tracer slurry was sprayed. A research technician ensured that the slurry was well mixed and then sprayed the subject with a pesticide hand sprayer by using a left to right sweeping motion (5 total passes) moving from the subject’s head to feet. The subject was then instructed to turn 180°, and this contamination process was repeated on the subject’s back. The fluorescent tracer slurry was developed after trying different combinations of commercially available oils, fluorescent products, and sprayers; the goal was the selection of a slurry that sprayed consistently, adhered to the PPE surface without runoff, and could be visualized under UV light.

Following contamination with the fluorescent tracer, subjects were then contaminated with the PSLs using a Collison nebulizer. Subjects were instructed to stand on another marked spot on the floor in the contamination room located 0.5 m from the outlet of the nebulizer. The nebulizer was on a stand placed at 1.5 m height. Subjects were instructed to face toward the nebulizer with palms forward, and then PSL nebulization began by supplying air to the aerosol generator (6 L/minute, 20 lbs per square inch gauge). The subjects were then asked to turn 90° every 60 seconds to ensure all surfaces of their external PPE were exposed to the nebulized PSLs. Following the 4-minute nebulization time, 2 high-efficiency particulate air filtration units (dry filter unit-1000, Lockheed Martin Integrated Systems, Gaithersburg, Maryland) were turned on operating at a combined 5000 L/minute and allowed to run for 5 minutes to fully purge the air of the contamination room prior to the subject exiting; the contamination room was approximately 25 m^3^ volume. An aerodynamic particle sizer (TSI, Shoreview, Minnesota) was included in the contamination room to monitor the particle number size distribution of the nebulized PSLs and the background levels of air contamination in between subjects. The average peak PSL concentration during contamination was 216 PSL/L air ± 488 PSL/L air. Past studies have shown that coughing can produce up to about 10^5^ particles/L air with a mean size by particle number of 1–2 µm [35]. Therefore, the PSL contamination in the present study is not outside of a realistic range for respiratory particulate that could be generated by a patient. For an illustration of the nebulized PSL particle number size distribution during subject contamination, see Supplementary Figure 1.

The subjects then doffed the PPE according to the CDC guidelines for care of patients with EVD including the use of a trained observer and doffing assistant [14]. The same individuals served in these roles for each participant, and the doffing assistant changed PPE between each participant to minimize the risk for subject cross-contamination. After doffing, the room was fully darkened, and a UV light was used to locate the areas for fluorescent tracer contamination. These were recorded on a standard sheet for each participant indicating locations and relative sizes of contaminated skin and scrub areas (Supplementary Figure 2). Finally, the subjects proceeded to the final station for PSL skin swabbing. The locations for skin swabbing (Table 3) were informed by subject matter expertise in healthcare, with past research indicating the most likely area for doffing self-contamination to be the hands, and the most common activity to result in self-contamination to be during removal of the PAPR hood [5, 7, 9, 11, 36, 37]. For more details on the derivation of skin surface area per swab location, see Supplementary Table 1.

**Table 3.** Locations for Skin Swabbing, Estimated Total Skin Surface Areas, and Respective Limits of Detection for Polystyrene Latex Spheres

Swab Location^a^	Description	Approximate Skin Surface Area^b^, cm^2^	Theoretical Limit of Detection for Swabbed Area^c^, No. of PSLs
Forehead	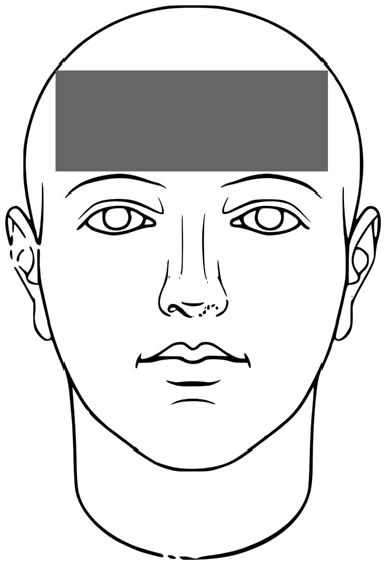	51.4	13
Right and left cheek	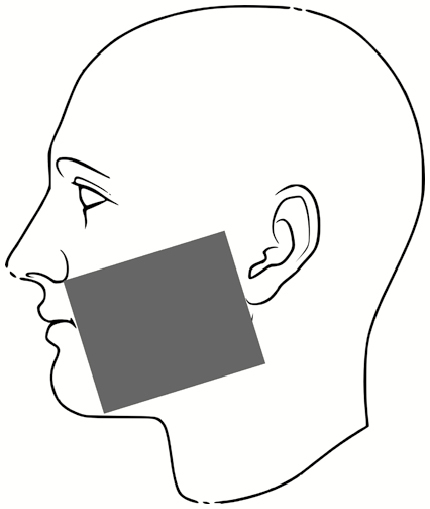	31.9	8
Chin	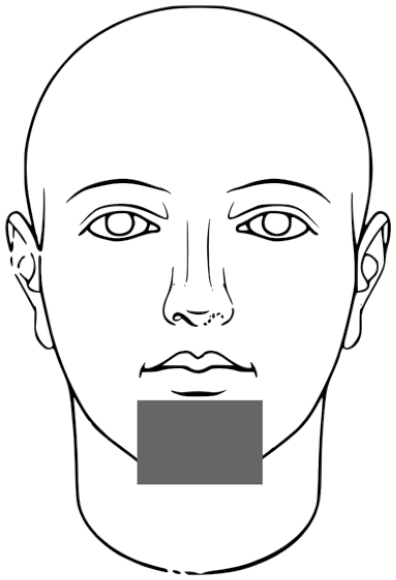	13.7	3
Right and left outer rim of ear	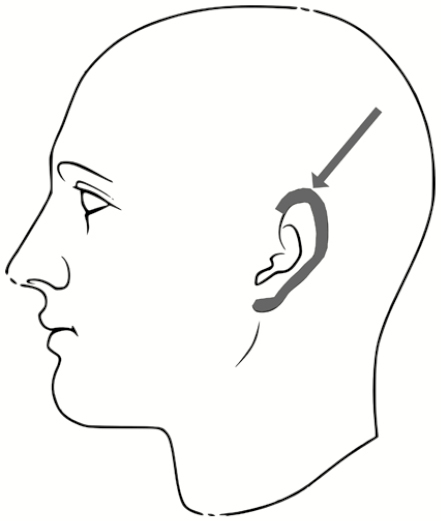	6.1	2
Right and left inner wrist	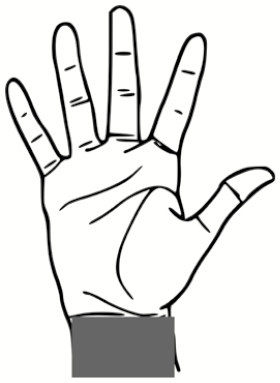	40.6	10
Right and left back of hand (fingers not included)	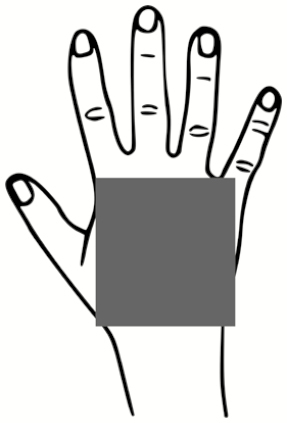	74.1	19
Index to thumb	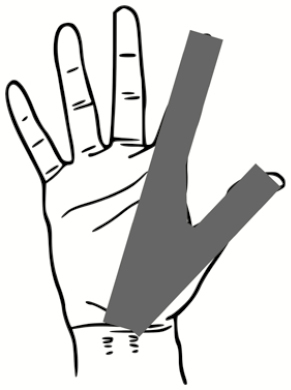	18.2	5

Abbreviation: PSL, polystyrene latex sphere.

^a^Where right and left samples are indicated, these samples were taken with a separate swab for right and left.

^b^For more details on the derivation of skin surface area per swab location, see Supplementary Table 1.

^c^Theoretical limit of detection (LOD) calculated using equation 1 and results presented in Table 3 indicating the overall recovery efficiency of PSL skin swabbing to be 40%, resulting in an LOD of 0.25 PSL/cm^2^.

## RESULTS

### PSL Protocols: Skin Swabbing and PSL Quantification by Microscopy

The overall average recovery efficiency for swabbing the PSLs from the porcine skin coupons was 40% ± 29% (Table 4). The precision, as indicated by the coefficient of variation (CV), ranged from 21% to 91% (Table 4). The CV tended to decrease as the spiked PSL number increased.

**Table 4.** Results Summary From Porcine Skin Swabbing to Recover and Detect Polystyrene Latex Spheres

Spiked No. of PSLs	Recovery, %, Average ± 1 SD	Coefficient of Variation, %	No. (%) of Skin Coupons With PSLs Detected
9 × 10^0^	60 ± 55	91	3/5 (60)
4 × 10^2^	24 ± 18	72	5/5 (100)
5 × 10^3^	49 ± 10	21	5/5 (100)
4 × 10^4^	33 ± 17	51	5/5 (100)
4 × 10^5^	32 ± 12	36	5/5 (100)

Abbreviations: PSL, polystyrene latex sphere; SD, standard deviation.

The theoretical limit of detection (LOD) for the PSL swabbing can be described as follows [24]:

$$\begin{array}{l} \;LOD\;\left(\frac{\#PSL}{cm^{2}}\right)=\;\frac{\left[\frac{1\;PSL\;x\;\left(\frac{total\;eluted\;volume}{filtered\;volume}\right)}{Swab\;recovery\;ef\;f\;iciency\;(\;\%\;)}\right]}{Swabbed\;skin\;area\;(cm^{2})} \end{array}$$

**Figure 2.**
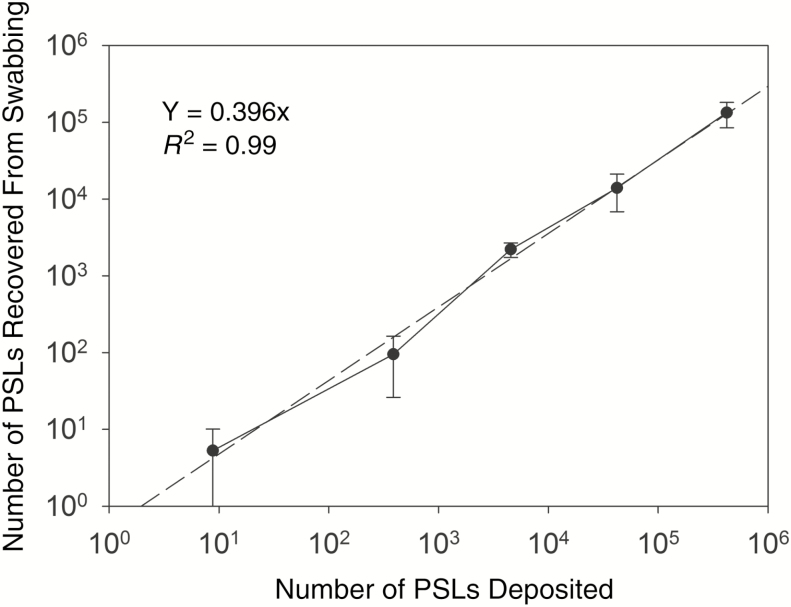
Linearity of recovery of polystyrene latex spheres (PSLs) after swabbing porcine skin coupons spiked with PSL numbers ranging from 10^0^ to 10^5^ (total of 25 swabbed skin coupons). Data are shown as average ± 1 standard deviation. Across the range of spiked PSL numbers, the average recovery efficiency was about 40% ± 29%. Abbreviation: PSLs, polystyrene latex spheres.

It is assumed that at least 1 PSL is required for detection as indicated in the equation above. For the present experiments, 1 mL of the 5-mL eluted swab suspension was filtered through the black polycarbonate filter. Assuming an average swab recovery efficiency of 40% and a swabbed surface area of 50 cm^2^ per porcine coupon, this results in a theoretical LOD of 0.25 PSL/cm^2^, or about 13 PSL per skin coupon. As illustrated in Figure 2, the number of PSLs recovered was found to be linear in relation to the number of PSLs applied to the skin coupon, with variability in swabbing recovery generally decreasing as the number of spiked PSLs increased. The high variability for the lowest number of spiked PSLs (~10) and the inability to detect PSLs on all 5 spiked coupons for this number support the validity of the theoretical LOD calculated above.

### Pilot Test: Skin Swabbing and Button Sampler Results

**Figure 3.**
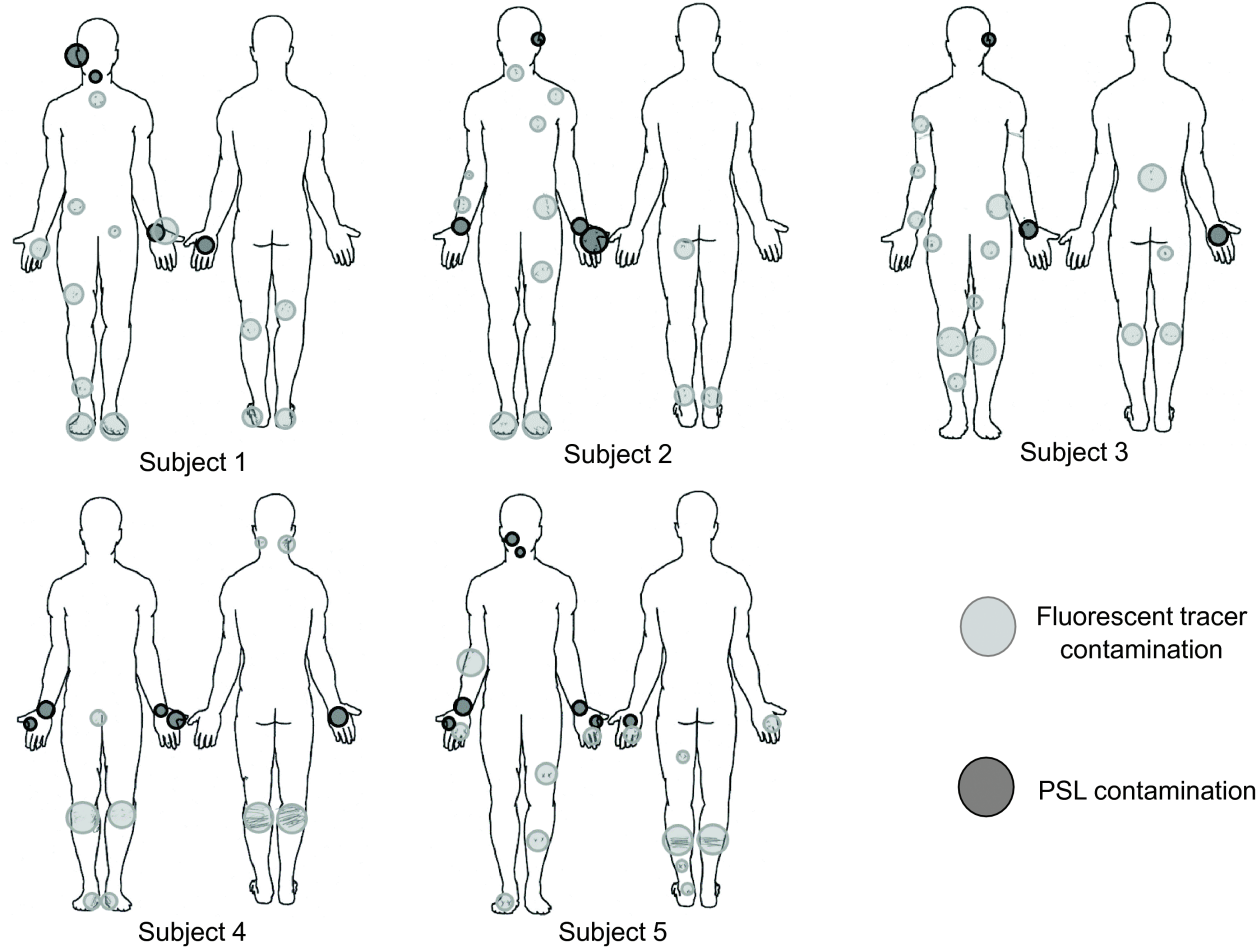
Qualitative illustrations of relative sizes and numbers of contamination spots for each subject for both types of contamination methods—fluorescent tracer method vs polystyrene latex sphere (PSL) method. The fluorescent tracer contamination was detected by eyesight under ultraviolet light, but the PSLs were swabbed and quantified using microscopy. PSLs were not detectable by eyesight alone. Abbreviation: PSL, polystyrene latex sphere.

All 5 of the subjects completed the full study protocol with an average doffing time of 8 minutes ± 2 minutes. Figure 3 illustrates the locations and relative sizes and numbers of contamination spots for each subject for the 2 types of contaminations tested in the pilot study. There are 3 apparent trends demonstrated by Figure 3: (1) All subjects were contaminated. The locations that were contaminated across every subject included the hands, face/head, and legs. (2) Only 2 of 5 subjects had overlapping detection of both types of contamination on a wrist (subject 1) and back/front of hands (subject 5). For all other subjects, the PSL method enabled a more sensitive means of skin contamination detection that was not detectable by the fluorescent tracer method alone. (3) The PSL method resulted in the identification of more skin contamination than the fluorescent method, while the fluorescent tracer method enabled detection of contamination on scrubs and shoes in addition to skin.

**Figure 4.**
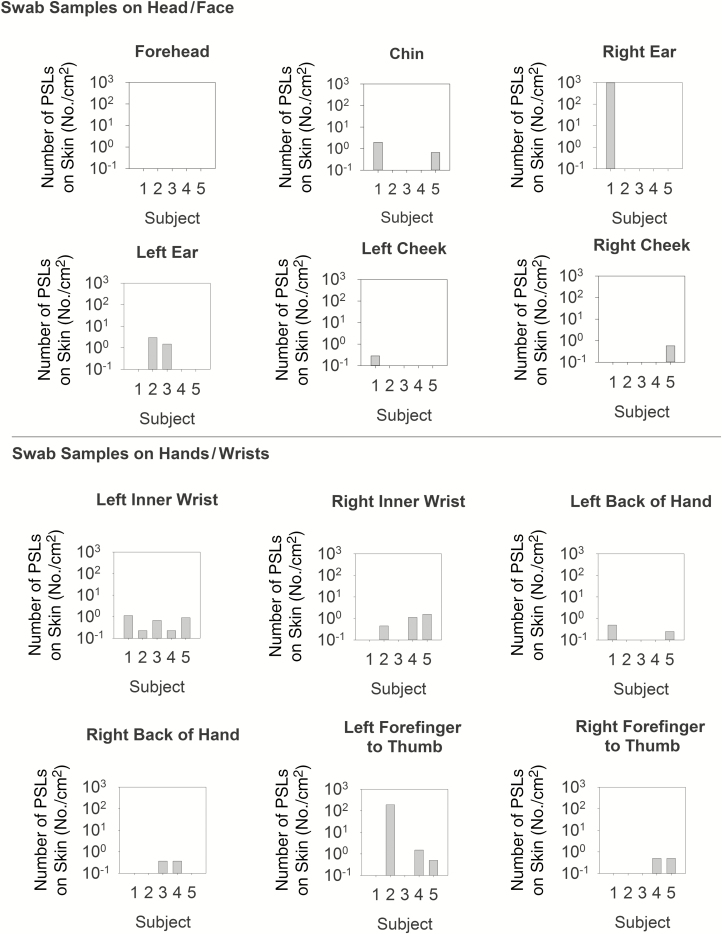
Skin swabbing results for polystyrene latex spheres (PSLs) detected across subjects and swab locations on the head/face vs hands/wrists after doffing of personal protective equipment. Results are presented as number of PSLs per cm^2^ of swabbed skin, as estimated using anthropomorphic data (Supplementary Table 1). Abbreviation: PSLs, polystyrene latex spheres.

Figure 4 illustrates the number of PSLs recovered from all of the skin swabbing locations for each subject; results are divided into 2 graphs (head/face vs hands/wrists) to better illustrate trends in contamination. Across all subjects, the hands/wrists were more commonly contaminated than areas of the head/face with 57% (17/30 swabs) vs 23% (7/30 swabs) of swabs resulting in PSL detection, respectively. For those swabs where PSLs were detected, the numbers ranged from 1 PSL/cm^2^ to 1390 PSL/cm^2^ on head/face, and 1 PSL/cm^2^ to 192 PSL/cm^2^ on hands/wrists. This highlights that the study subjects varied greatly in individual tendencies for self-contamination.

**Figure 5.**
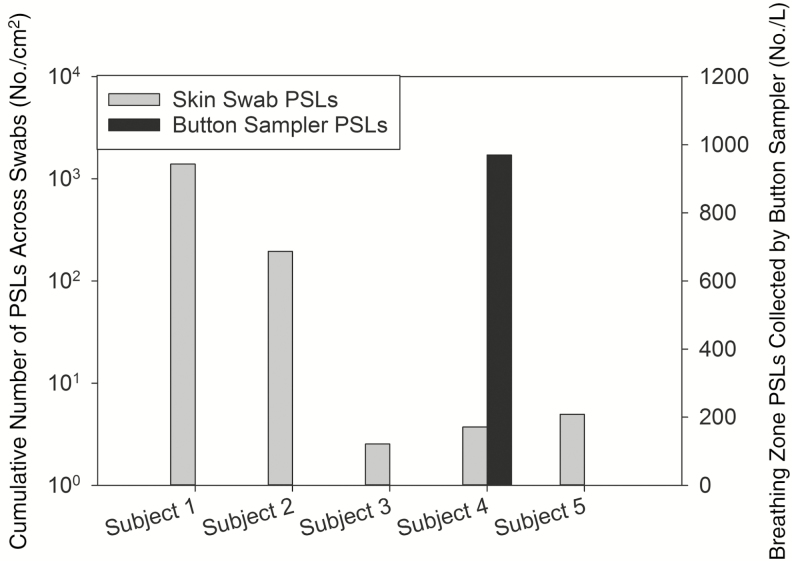
Cumulative number of polystyrene latex spheres (PSLs) across each subject’s skin swabs vs the concentration of PSLs measured in the breathing zone of study subjects via Button Sampler during personal protective equipment doffing. Only subject 4 had any PSLs detected in the breathing zone. Abbreviation: PSLs, polystyrene latex spheres.

While all subjects had varying levels of PSL skin contamination, only subject 4 had PSLs detected in the breathing zone as indicated by the Button Sampler; given that subject 4 had relatively low levels of skin PSL contamination, there does not appear to be a clear relationship between skin contamination and breathing zone presence of PSLs (Figure 5).

## DISCUSSION

The results obtained in the present study for porcine skin swabbing recovery efficiencies of PSLs (40% ± 29%) are similar to the results reported for previous studies investigating the recovery efficiency of *Bacillus anthracis* spores from stainless steel coupons using premoistened macro-foam swabs. Spiking spore numbers of 10^0^ to 10^4^ and eluting swabs with a 2-minute vortex, average recovery efficiencies have been reported ranging from 16% to 64% with overall averages typically in the range of 25%–45% [22, 24, 38]. The precision for the PSL recovery efficiency (CV = 21%–91%) was also similar to previous studies investigating the same range of spiked *B. anthracis* spore numbers from steel coupons (21%–91%) [38]. However, it should be noted that, as opposed to steel coupons, the porcine skin coupons used in the present study were inherently flawed with minor imperfections, such as skin wrinkles and divots [39], likely adding to the sampling variability. Regardless, the use of skin coupons is a better representation of actual skin swabbing performed in the pilot test.

As the PSLs simulate a microorganism-sized particle, the PSL method described herein allows for quantification of the actual total number of potential pathogens that could be expected to be present on skin after doffing [13]. The PSL contamination method simulates a realistic level of contamination that a healthcare worker might experience from a coughing patient with a respiratory pathogen [35]. This has implications not only for evaluating contamination from doffing practice, but for assessing possible areas of high risk for leading to infection in a real patient care environment. Recent studies have also highlighted the importance of including a contamination technique that allows for quantitative measures of doffing self-contamination, with a means to relate this information to real-world potential for infection; specifically, these studies have incorporated nonpathogenic viruses (bacteriophages MS2 and Φ6) into study protocols as part of the fluorescent tracer mix to compare virus contamination results to the fluorescent tracer [5, 37, 40]. However, this type of protocol incorporating nonpathogenic viruses may be logistically impractical in all clinical environments.

While every subject was contaminated to some extent, only 2 of 5 subjects had overlapping detection of both types of contamination (ie, PSL and fluorescent tracer) on a body part. The PSL method did enable a more sensitive means of skin contamination detection, but this new method cannot be used to universally scan scrubs for contamination; therefore, it serves as a complement to the fluorescent tracer method to study self-contamination during PPE doffing. The wide variability in the tendencies for the study subjects to contaminate themselves highlights the individualized aspect of this type of research. PPE doffing educational and training programs should therefore include individual feedback to healthcare workers to reduce overall levels of contamination and to identify and provide additional training for individuals who are at higher risk for self-contamination [9].

The finding that one of the study subjects had PSLs in their breathing zone during doffing warrants further investigation. The occurrence of PSL air contamination may be attributed to the doffing style used by this subject, and this will be explored further in a larger, future study. With an airborne PSL concentration of 9.7 × 10^5^ PSL/L air, and assuming a mean moderate intensity, short-term inhalation rate of 27 L/minute for adults [41] with an average doffing time of 8 minutes, this would result in exposure to >2 × 10^8^ PSLs. However, this is assuming that the PSL concentration was consistent throughout doffing. A limitation of the present study is that the use of only 1 Button Sampler throughout the contamination and doffing procedures to measure breathing zone PSLs results in an inability to distinguish when during these processes the exposures may have occurred. Regardless, the potential for a high level of inhalational exposure during PPE doffing warrants further investigation. Future work will include a more thorough investigation of the potential for PSLs to be present in the doffer’s breathing zone by a series of filter samplers that turn on sequentially during PPE doffing to identify the doffing steps that introduce the most risk. These samplers will also be placed in a grid around the doffer to characterize the PSL concentration throughout the space with fans to ensure a well-mixed study room.

## CONCLUSIONS

Overall, this study provides a well-characterized method that can be used to quantitate levels of skin and inhalational contact with simulant pathogen particles. Cross-comparison of the results obtained from this new PSL method vs the traditional fluorescent tracer method suggests that future studies should use both contamination methods as complementary approaches to allow for more thorough, comprehensive, and objective contamination assessment. Further research will be required to better understand the presence of airborne particles in the breathing zone during PPE doffing, and how that may or may not represent true healthcare worker exposure.

## Supplementary Data

Supplementary materials are available at *Clinical Infectious Diseases* online. Consisting of data provided by the authors to benefit the reader, the posted materials are not copyedited and are the sole responsibility of the authors, so questions or comments should be addressed to the corresponding author.

ciz616_suppl_Supplementary_Information

## Notes

***Acknowledgments.*** The authors thank contributing members of the Johns Hopkins EpiCenter team for their assistance in this project, and all of our volunteer participants.

***Financial support.*** This work was supported by funding from the Prevention Epicenters Program of the Centers for Disease Control and Prevention (cooperative agreement 1U54CK000447).

***Supplement sponsorship.*** This article appears as part of the supplement “Personal Protective Equipment for Preventing Contact Transmission of Pathogens: Innovations from CDC’s Prevention Epicenters Program,” sponsored by the CDC’s Prevention Epicenters Program.

***Potential conflicts of interest.*** D. D. and K. R. L. have a patent pending (“Method for Release and Tracking of Aerosol Particles Within a Hospital Environment”). All other authors report no potential conflicts of interest. All authors have submitted the ICMJE Form for Disclosure of Potential Conflicts of Interest. Conflicts that the editors consider relevant to the content of the manuscript have been disclosed.
