# Supplementary material for: Development and Comparison of Complementary Methods to Study Potential Skin and Inhalational Exposure to Pathogens During Personal Protective Equipment Doffing
Source: Clin Infect Dis. 2019 Sep 13;69(Suppl 3):S231–40. doi: 10.1093/cid/ciz616 (PMC6761368; doi:10.1093/cid/ciz616)
Supplement: ciz616_suppl_Supplementary_Information [file ciz616_suppl_supplementary_information.docx]

**
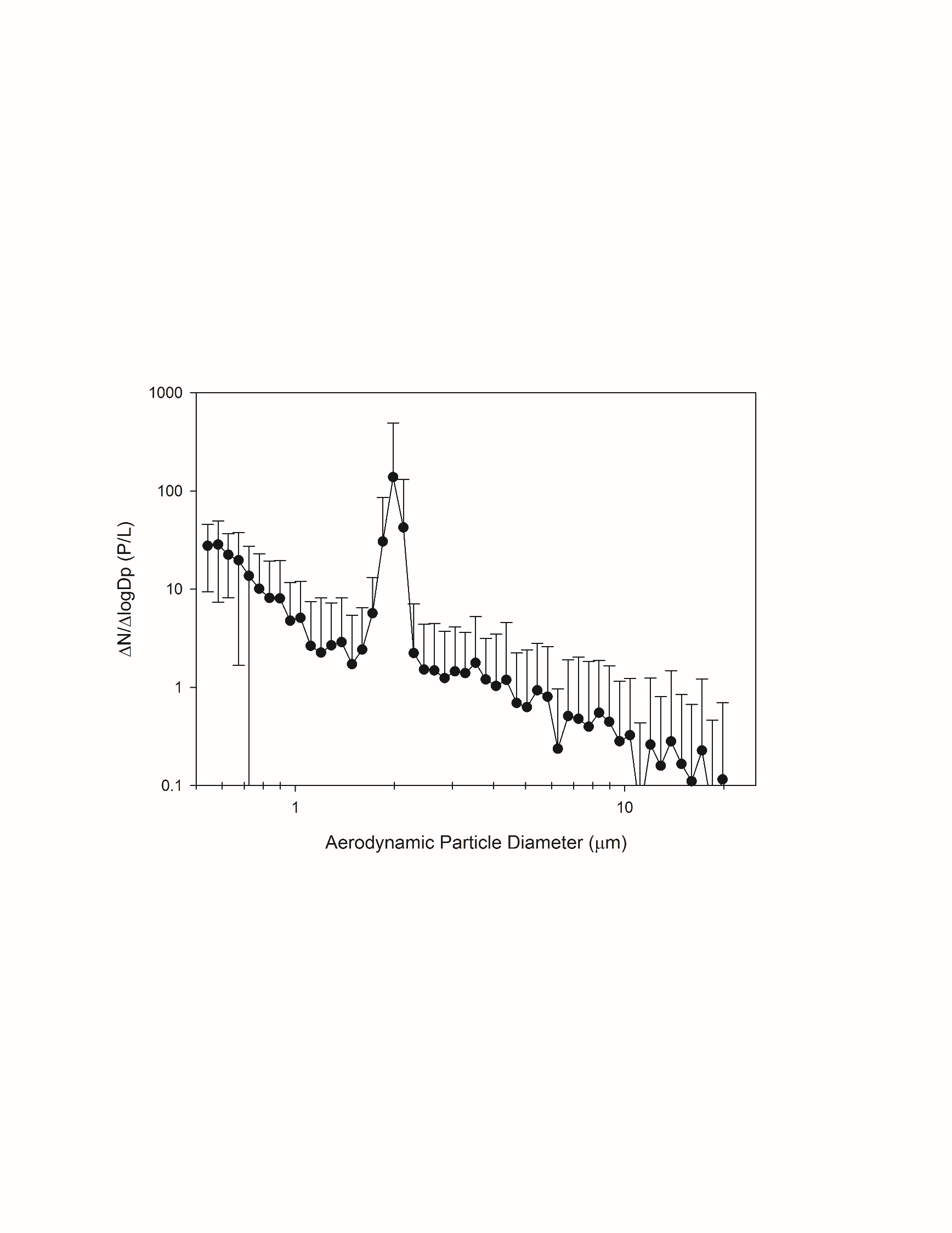
**

**Figure S1.** Particle number size distribution measured in the contamination room by Aerodynamic Particle Sizer (TSI Inc., Shoreview, MN) during subject contamination with the 2 µm sized polystyrene latex spheres.


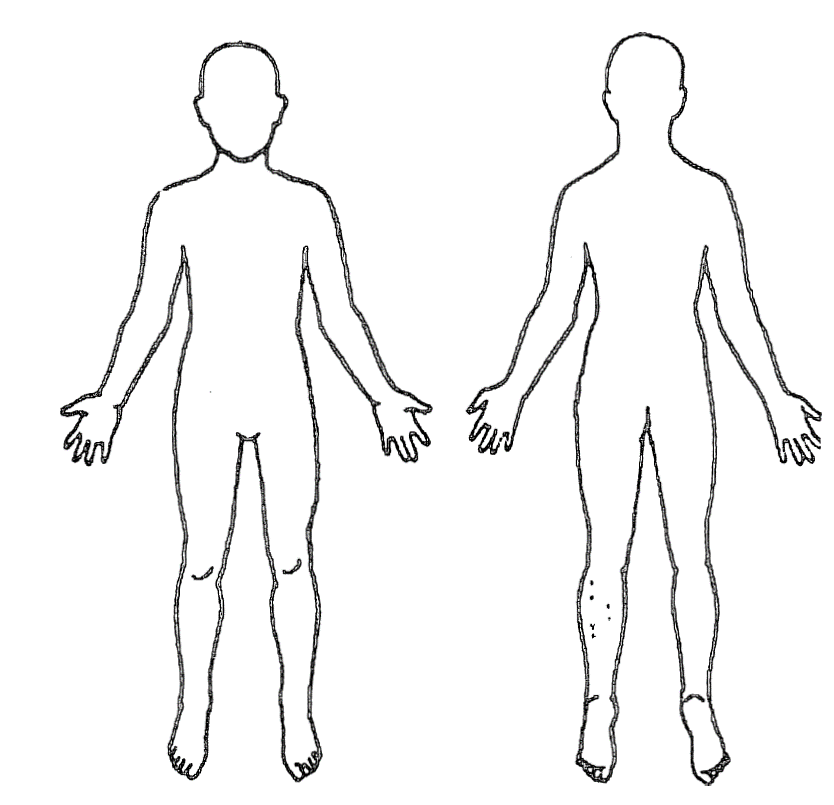


**Figure S2.** Example blank sheet to record participant contamination locations.

| Table S1. Details on the Swabbed Skin Locations and Derivations of Estimated Skin Surface Areas | | | | | | |
| --- | --- | --- | --- | --- | --- | --- |
| Swab Location ^I^ | **Landmarks and Description of Swabbed Skin Area** | | **Approximate Skin Surface Area (cm^2^)** | **Theoretical Limit of Detection for Swabbed Area ^II^** | **Anthropomorphic Measurements ^III^** | |
|  | **Height** | **Width** |  |  | **References** | **Number of Measurements by Gender (M, F or Both) Represented from Referenced Studies** |
| Forehead | Glabella to trichion  (above eyebrows to hairline) | Biocular breadth; distance between outer corners of the eyes | 51.4 | 13 | [1-5] | Biocular breadth:  Female: N = 283  Male: N = 228  Both: N = 185  Glabella-trichion:  Female: N = 158  Male: N = 72 |
| Right and left cheek | Menton to subnasion  (bottom of chin to bottom of nose) | [Bizygomatic breadth – cheilion-cheilion]/2  (One cheek width = face width minus width of mouth divided by two) | 31.9 | 8 | [1, 2, 4, 6-11] | Cheilion-cheilion:  Female: N = 2154  Male: N = 2822  Both: N = 155  Bizygomatic breadth:  Female: N = 3857  Male: N = 4488  Both: N = 461  Menton-subnasion:  Female: N = 2208  Male: N = 1774  Both: N = 461 |
| Chin | Sublabiale to gnathion  (vertical height of the chin) | Cheilion to cheilion  (width of mouth) | 13.7 | 3 | [1, 2, 4, 5, 7, 8, 11] | Cheilion-cheilion:  Female: N = 2154  Male: N = 2822  Both: N = 155  Sublabiale-gnathion:  Female: N = 50  Male: N = 50  Both: N = 185 |
| Right and left outer rim of ear | Circumference of the outer ear using measurements of ear length and width to estimate ellipse | Thickness of ear skin and cartilage | 6.1 | 2 | [4, 7, 12-17] | Ear height:  Female: N = 1133  Male: N = 1343  Both: N = 270  Ear width:  Female: N = 391  Male: N = 435  Ear skin and cartilage thickness:  Both: N = 47 |
| Right and left inner wrist | Half circumference of wrist | From wrist crease line extending down arm 5 cm | 40.6 | 10 | [18, 19] | Wrist circumference:  Female: N = 84  Male: N = 192 |
| Right and left back of hand (fingers not included) | Half total hand length | Hand breadth | 74.1 | 19 | [10, 18, 20] | Hand length and width:  Female: N = 2095  Male: N = 4353 |
| Index to thumb | Length of hand plus wrist to thumb-tip length | Width of swab head | 18.2 | 5 | [10] | Hand length and wrist to thumb-tip length:  Female: N = 2095  Male: N = 4353 |
| Notes:  I – Where right and left samples are indicated, these samples were taken with a separate swab for right and left.  II – Theoretical limit of detection (LOD) calculated using equation 1 and results presented in Table 3 indicating the overall recovery efficiency of PSL skin swabbing to be 40% resulting in an LOD of 0.25 PSL/cm^2^.  III – The median was the preferred value to extract from all referenced studies. Where only a mean was provided instead, then this was selected as the extracted measurement from the study. For studies that presented data related to health status, only the measurements from health controls were used. All included studies were selected to represent males and females, and only adult measurements with included. | | | | | | |

**References**

1. Milutinovic J, Zelic K, Nedeljkovic N. Evaluation of Facial Beauty Using Anthropometric Proportions. The Scientific World Journal **2014**; 2014: 428250.

2. Asghari A, Rajaeih S, Hassannia F, et al. Photographic facial soft tissue analysis of healthy Iranian young adults: anthropometric and angular measurements. Med J Islam Repub Iran **2014**; 28: 49-.

3. Reksodiputro MH, Koento T, Boedhihartono, Sclafani AP. Facial anthropometric analysis of the javanese female. Arch Facial Plast Surg **2009**; 11(5): 347-52.

4. Bozkir MG, Karakas P, Oguz Ö. Vertical and horizontal neoclassical facial canons in Turkish young adults. Surg Radiol Anat **2004**; 26(3): 212-9.

5. Aung SC, Ngim RCK, Lee ST. Evaluation of the laser scanner as a surface measuring tool and its accuracy compared with direct facial anthropometric measurements. Br J Plast Surg **1995**; 48(8): 551-8.

6. Young J. Head and face anthropometry of adult U.S. citizens. In: Office of Aviation Medicine FAA. Washington DC: US Department of Transportation **1993**.

7. Choe KS, Sclafani AP, Litner JA, Yu G, Romo T, Iii. The korean american woman&#39;s face: Anthropometric measurements and quantitative analysis of facial aesthetics. Arch Facial Plast Surg **2004**; 6(4): 244-52.

8. Farkas LG, Katic MJ, Hreczko TA, Deutsch C, Munro IR. Anthropometric proportions in the upper lip-lower lip-chin area of the lower face in young white adults. Am J Orthod Dentofacial Orthop **1984**; 86(1): 52-60.

9. Yang LEI, Shen H, Wu GAO. Racial Differences in Respirator Fit Testing: A Pilot Study of Whether American Fit Panels are Representative of Chinese Faces. The Annals of Occupational Hygiene **2007**; 51(4): 415-21.

10. Claire Gordon, Thomas Churchill, Charles Clauser, et al. 1988 Anthropometric survey of US army personnel: Summary statistics interim report. In: Research DaEC. Natick, MA: United States Army Natick, **1989**.

11. Zhuang Z, Bradtmiller B, Friess M. Head-and-face anthropometric survey of U.S. respirator users. J Occup Environ Hyg **2005**; 2(11): 567-76.

12. Burkhard MD, Sachs RM. Anthropometric manikin for acoustic research. The Journal of the Acoustical Society of America **1975**; 58(1): 214-22.

13. Brucker MJ, Patel J, Sullivan PK. A Morphometric Study of the External Ear: Age- and Sex-Related Differences. Plast Reconstr Surg **2003**; 112(2): 647-52.

14. Bozkır MG, Karakaş P, Yavuz M, Dere F. Morphometry of the External Ear in Our Adult Population. Aesthetic Plast Surg **2006**; 30(1): 81-5.

15. Wang B, Dong Y, Zhao Y, Bai S, Wu G. Computed tomography measurement of the auricle in Han population of north China. J Plast Reconstr Aesthet Surg **2011**; 64(1): 34-40.

16. Alexander KS, Stott DJ, Sivakumar B, Kang N. A morphometric study of the human ear. J Plast Reconstr Aesthet Surg **2011**; 64(1): 41-7.

17. Danter J, Siegert R, Weerda H. Ultrasound measurement of skin and cartilage thickness in healthy and reconstructed ears with a 20-MHz ultrasound device. Laryngo-Rhino-Otologie **1996**; 75(02): 91-4.

18. NASA. Anthropometry and Biomechanics. Vol. 1. Houston, TX: National Aeronauthics and Space Administration, Johnson Space Center, **2018**:32-79.

19. Moghtaderi A, Izadi S, Sharafadinzadeh N. An evaluation of gender, body mass index, wrist circumference and wrist ratio as independent risk factors for carpal tunnel syndrome. Acta Neurol Scand **2005**; 112(6): 375-9.

20. Abdel-Malek AK, Ahmed AM, El Aziz El Sharkawi SA, El Maksoud Abd El Hamid NA. Prediction of stature from hand measurements. Forensic Sci Int **1990**; 46(3): 181-7.
